# Brain injury biomarkers as targets for drugs development and personalized treatment for traumatic brain injury patients

**DOI:** 10.3389/fphar.2025.1606174

**Published:** 2025-05-26

**Authors:** Melissa Sandler, Sulaiman Almohaish, Gretchen M. Brophy

**Affiliations:** ^1^ Department of Pharmacotherapy and Outcomes Science, School of Pharmacy, Virginia Commonwealth University, Virginia, United States; ^2^ College of Clinical Pharmacy, King Faisal University, Al-Ahsa, Saudi Arabia

**Keywords:** biomarkers, traumatic brain injury, drug development, drug discovery, pharmacology

## Abstract

Drug treatment protocols for traumatic brain injury (TBI) that result in long-term, positive outcomes have yet to be determined for various reasons, including diversity of injury and difficulty in measuring outcomes. Brain injury biomarkers are increasingly being used for drug development and treatment research in patients with TBI to supplement pharmacokinetic studies, provide evidence of drug mechanism of action, detect early and long-term clinical outcomes, and homogenize study populations. The use of biomarkers to influence TBI drug development and treatment trials has the potential to lead to more innovative research and personalized patient care. Future TBI clinical trials that utilize these innovative biomarkers study designs and demonstrate strong correlations between biomarkers and clinical outcomes could permit shorter, less expensive, and more successful clinical trials.

## 1 Introduction

Biomarkers are measurable indicators of biological processes. They vary from molecular to histologic to radiographic markers and have the potential to augment prognostication, diagnosis, and monitoring of patients with various disease states. Importantly for drug development and treatment trials, biomarkers have the potential to be used to measure response for both safety and efficacy endpoints. Biomarkers may decrease the cost and increase efficiency of the drug treatment studies by informing researchers of drug response and toxicity in preclinical and early phase clinical studies (FDA, 2020). This early indication may lead to more rapid determination of drug safety and effectiveness in combination with clinical outcome measures in research trials as well as decrease research-associated costs (Kochanek et al., 2020).

For traumatic brain injury (TBI), biomarkers have increased prognostic capabilities, improved clinical resource utilization, and directed researchers to new drug targets (Wang et al., 2018; Wang et al., 2021). Using biomarkers to guide drug development or treatment of patients with TBI is particularly appealing because little to no progress has been made in the identification of therapies that truly target the pathophysiologic mechanisms of TBI. Additionally, the heterogeneity of patients with TBI makes a single treatment for all patients with TBI unlikely. Biomarkers have the potential to identify patients with similar mechanisms of injury and to target phenotypes likely to respond to specific treatment strategies. Although early objective outcomes, including mortality and acute functional status, are important, the long-term impacts of TBI such as loss of productivity, loss of independence, and prolonged neurological dysfunction are extremely important quality of life measures for those who survive the initial injury. If a biomarker is found to correlate strongly with these long-term TBI outcomes, a targeted approach for drug development and treatment in the acute period after injury may be possible.

Brain injury biomarkers currently under investigation in patients with TBI have been described and categorized based on physiology in previous publications (Edalatfar et al., 2021). The three most common categories were cytokines, coagulation parameters, and nerve tissue proteins. Nerve tissue proteins include, but are not limited to, S100β, glial fibrillary acidic protein (GFAP), microtubule-associated proteins (MAP), neurofilament light chain proteins (NF-L), and myelin basic proteins (MBP). It has also been postulated that biomarkers can differentiate type of brain injury [e.g., phosphorylated axonal neurofilament heavy chain (pNf-H) indicates axonal injury and ubiquitin C-terminal hydrolase-L1 (UCH-L1) suggests neuronal cell body injury] (Table 1). (Wang et al., 2018; Gutierre et al., 2020)

**TABLE 1:** Traumatic brain injury biofluid biomarker characteristics.

Biofluid biomarker	Injury type	Acuity^a^	Drug impact
Glial fibrillary acidic protein (GFAP)	Astrocytes	Acute	Decreased by: cyclosporin A Not impacted by: epoetin alfa, metformin, probenecid plus N-acetylcysteine, progesterone
Inflammatory markers: C-reactive protein (CRP), interleukin (IL)	Systemic markers of autoimmunity or inflammation/neuroinflammation	Subacute/chronic	Decreased by: metformin Not impacted by: cyclosporin A, vitamin D
Microtubule-associated proteins (MAP)	Dendrites	Subacute	
Myelin basic protein (MBP)	Axons	Subacute	Decreased by: hypertonic saline in dextran
Neurofilament proteins (NF)	Axons	Subacute	Decreased by: cyclosporin A Not impacted by: epoetin alfa
Neuron-specific enolase (NSE)	Neuronal cell body	Acute	Decreased by: hypertonic saline in dextran, memantine Not impacted by: L-carnitine, probenecid plus N-acetylcysteine
S100β	Glial cells, astrocytes	Acute	Decreased by: hypertonic saline in dextran, metformin Not impacted by: epoetin alfa, progesterone
Spectrin breakdown product 120 (SBDP120)	Apoptosis	Subacute	
SBDP145/150	Necrosis	Acute	Not impacted by: progesterone
Tau	Axons	Chronic	Decreased by: cyclosporin A
Ubiquitin C-terminal hydrolase-L1 (UCH-L1)	Neuronal cell body	Acute	Decreased by: cyclosporin A Not impacted by: epoetin alfa, progesterone

^a^ Acute: ∼ minutes-hours; subacute: ∼ hours-days; chronic: ∼ weeks-months/years.

The use of biomarkers in TBI drug development and treatment research is rapidly growing. This article aims to describe how biomarkers may be used as drug targets to augment the efficiency and effectiveness of drug development and treatment studies for the treatment of patients with TBI.

## 2 Biomarker applicability in traumatic brain injury clinical studies

Most pharmacological TBI clinical trials to date have incorporated biomarkers as a secondary endpoint to describe the effect of the drug on injury mechanisms (Tables 1, 2). For example, two clinical studies of treatment with erythropoietin found that biomarker concentrations and profiles were not affected by erythropoietin, suggesting that this drug does not impact the pathophysiologic processes of TBI in the population studied (Hellewell et al., 2018; Hellewell et al., 2020). Trials of hypertonic saline-dextran solution reported reductions in various inflammatory markers, as well as in S100β and neuron-specific enolase (NSE), which correlated with CT findings and clinical outcomes (Baker et al., 2009; Rhind et al., 2010). As including biomarker concentrations becomes more commonplace, the correlation, or lack thereof, between biofluid biomarker concentrations, pharmacokinetics and pharmacodynamics of potential neuroprotective drugs, and clinical outcomes will become more apparent.

**TABLE 2:** Select TBI biomarker clinical trials with pharmacologic intervention.

	Population	Intervention	Outcome
Cyclosporine			
Kelsen, et al., 2019 [Copenhagen Head Injury Ciclosporin (CHIC)] (Kelsen et al., 2019)	N = 16 Severe TBI	Novel parenteral lipid emulsion formulation of cyclosporine 2.5 mg/kg loading dose then CIVI x 5 days (5 or 10 mg/kg/day)	CSF concentrations of GFAP (slope −5.80 during infusion vs 5.84 post-infusion, p = 0.0061), Nf-L (slope −0.17 during infusion vs. 0.27 post-infusion, p = 0.0171), tau (slope −0.05 during infusion vs. 0.11 post-infusion, p = 0.0266), and UCH-L1 (slope −1.02 during infusion vs. 1.73 post-infusion, p = 0.0017) consistently decreased during treatment and then rose when CIVI was stopped
Mazzeo et al. (2006)	N = 49 Severe TBI	Cyclosporin A 5 mg/kg/day IV over 24 h once or twice after a 24 h washout or placebo within 12 h of injury	Cyclosporin A did not impact total absolute lymphocyte count, CD3^+^, mature T cells, CD4^+^, CD8^+^
Epoetin Alfa			
Hellewell, et al., 2018 (Subgroup of Australian EPO-TBI) (Hellewell et al., 2018)	N = 44 Moderate to severe TBI	Epoetin alfa 40,000 units SC vs placebo within 24 h of injury and on days 8 and 15	Erythropoietin did not decrease UCH-L1 (AUC 135.4 vs 194.6, p = 0.72) or pNf-H (AUC 457.6 vs. 199.9, p = 0.44) in first 6 days - consistent with lack of improved clinical outcome (unfavorable GOS-E in 39% of treatment group vs. 38% placebo)
Hellewell, et al., 2021 (Subgroup of Australian EPO-TBI) (Hellewell et al., 2020)	N = 44 Moderate to severe TBI	Epoetin alfa 40,000 units SC vs placebo within 24 h of injury and on days 8 and 15	Erythropoietin did not decrease GFAP or S100β in first 6 days - consistent with lack of improved clinical outcome (unfavorable GOS-E in 39% of treatment group vs. 38% placebo)
Hypertonic Saline			
Baker et al. (2009)	N = 64 Severe TBI	250 mL of 7.5% hypertonic saline in 6% dextran 70 (HTS) vs. 250 mL of normal saline (NS) pre-hospital, within 4 h of injury	S100β concentrations were twofold lower and NSE concentrations threefold lower in HTS vs. NS groups at admission MBP increased in NS group at hour 48, but not HTS group
Rhind et al. (2010)	N = 65 Severe TBI	250 mL of 7.5% hypertonic saline in 6% dextran 70 (HTS) vs 250 mL of normal saline (NS) pre-hospital, within 4 h of injury	HTS blunted release of some inflammatory and prothrombotic markers including CD62L, CD11b, CD66b, sE-selectin, sVCAM-1, TNF-α, IL-10, and D-dimer, but not others (CD63, sL-selectin, sICAM-1, sTM)
Probenecid and N-acetylcysteine			
Clark et al. (2017)	N = 14 Severe TBI Pediatrics	Probenecid 25 mg/kg pFT load then 10 mg/kg every 6 h x 11 doses + NAC 140 mg/kg load pFT then 70 mg/kg every 4 h x 17 doses versus placebo within 24 h of injury	No difference in NSE (p = 0.441) or GFAP (p = 0.596) serum concentrations
Hagos et al. (2018)	N = 12 Severe TBI Pediatrics	Probenecid 25 mg/kg pFT load then 10 mg/kg every 6 h x 11 doses + NAC 140 mg/kg load pFT then 70 mg/kg every 4 h x 17 doses versus placebo within 24 h of injury	Glutathione concentrations higher in the treatment group than the placebo group
Other Drugs			
Mahmoodpoor et al. (2018)	N = 40 Severe TBI	L-carnitine 1 g pFT every 12 h × 7 days vs. placebo within 24 h of injury	No difference in NSE concentrations between groups
Mokhtari et al. (2018)	N = 41 Moderate TBI	Memantine 30 mg PO or pFT every 12 h × 7 days vs standard care within 24 h of injury	NSE lower in the memantine group on day 7 (5.03 ± 3.25 and 10.04 ± 5.72 ng/mL, p = 0.003)
Taheri et al. (2019)	N = 30 Severe TBI	Metformin 1 g pFT every 12 h × 5 days vs usual management within 48 h	S100β and neutrophil to lymphocyte ratio declined significantly in metformin vs control group (p < 0.001 and 0.017, respectively; no difference in GFAP concentrations (p > 0.05)
INTREPID-2566 2018 (Neuren Pharmaceuticals Limited, 2018)	N = 261 Moderate to severe TBI	NNZ-2566 (Trofinetide) 20 mg/kg IV over 10 min then CIVI at various doses x 72 h versus placebo infusion	Results not reported; GFAP and UCH-L1 planned secondary outcomes
Patel, et al., 2017 (DASH After TBI) (Patel et al., 2012)	N = 48 Severe TBI	Propranolol 1 mg IV every 6 h + clonidine 0.1 mg pFT every 12 h vs. placebo within 48 h of injury	Plasma norepinephrine concentrations, ng/mL [median (IQR)]: treatment 962 (508–1,471) vs. placebo 714 (391–1,257)
Sharma et al. (2020)	N = 35 Moderate to severe TBI	Vitamin D 120,000 IU pFT once vs placebo within 24 h	No differences in IL-6 (p = 0.08), IL-2 (p = 0.36), IFN-*γ* (p = 0.65) concentration in treatment vs placebo groups Decrease in TNF-α concentration (p = 0.02)
Korley, et al., 2021 (BIO-ProTECT, *post hoc* analysis of PROTECT III trial) (Korley et al., 2020)	N = 566 Moderate to severe TBI	Progesterone IV loading dose, CIVI, and taper x 96 h total in lipid emulsion versus placebo within 4 h of injury	No difference in GFAP, UCHL-1, S100β, or SBDP150 at 24 or 48 h post-injury (p > 0.15 for all comparisons)
Masbough et al. (2024)	N = 35 Moderate to severe TBI	Vitamin D 300,000 IU IM once vs no intervention	Lower IL-1β (p = 0.03) in the Vitamin D group than control group, but no difference in IL-6; GOS-E at 3 months higher in Vitamin D group

### 2.1 Surrogate biomarkers in combination with pharmacokinetic parameters

Although there are many reasons a drug may fail in clinical trials, one explanation may be that the drug did not achieve therapeutic concentrations at the site of action to achieve the desired pharmacodynamic effect. Currently, drug pharmacokinetic parameters are assessed to determine what doses will produce the concentrations needed to achieve the therapeutic effect observed in preclinical trials. There are multiple pitfalls in using pharmacokinetic parameters in this way. For one, although trials often report the drug concentration achieved, this is hard to interpret because therapeutic CSF or blood concentration ranges are not known for most drugs. Additionally, although assays are available for some drugs to measure concentrations, most drugs do not have commercially available assays. Lastly, even if an assay is available, it is not always feasible to collect a sample from the site of action (e.g., CSF from the brain or brain tissue); therefore, it is not known if the drug reached the site of action.

Biomarker concentrations as a surrogate for drug concentrations could be more clinically relevant than a drug concentration if the biomarker concentration correlates closely with clinical outcomes. Like traditional dose-finding studies, the therapeutic dose of a drug could be determined by the largest decrease or increase in a biomarker suggesting that it had the largest pharmacodynamic effect. Using biomarkers in this way would decrease the need for the development of a multitude of drug assays as a single biomarker assay could be used for multiple drugs acting at the same site.

Another obstacle that may be overcome by using biomarkers in this way is the translation from animal models to humans. Currently, body surface area or weight based dosing is used to estimate the effective dose in humans; instead, change in biomarker concentration may more effectively identify an effective dose.

The phase I study of probenecid plus NAC illustrates the use of known drug pharmacokinetic parameters in combination with a surrogate biomarker (Hagos et al., 2018; Clark et al., 2017). Due to concern for NAC reaching therapeutic concentrations at its site of action in the CSF, it was administered in combination with probenecid to decrease active transport out of the brain. Although the study directly measured drug concentrations in both the blood and CSF, the *post hoc* analysis measured glutathione concentrations in the CSF which should increase when NAC is administered. Because these CSF concentrations were increased in the treatment group, the authors concluded that NAC was achieving therapeutic concentrations in the brain. Future studies can use this method of measuring a surrogate biomarker known to be impacted by the drug being studied to ensure that effective drug concentrations are achieved at the site of action.

The Decreasing Adrenergic or Sympathetic Hyperactivity After Traumatic Brain Injury (DASH after TBI) trial illustrates the idea of measuring pharmacodynamic effect as well (Patel et al., 2012). Propranolol and clonidine were administered to patients with severe TBI to block detrimental sympathetic storming associated with high catecholamine concentrations which translates to poor clinical outcomes. Rather than measuring drug concentrations, norepinephrine concentrations were compared to assess pharmacodynamic effect. The combination of propranolol and clonidine was not shown to decrease norepinephrine concentrations compared to placebo which aligned with lack of difference in the clinical primary outcome of ventilator-free days. Although the lack of change in norepinephrine concentrations could have been related to this drug combination being ineffective, there are other factors to consider, such as sample timing and rapid norepinephrine degradation, that influence norepinephrine concentrations.

### 2.2 Evidence of drug mechanism of action

Biomarkers may provide indications as to the mechanism by which drugs achieve a clinical benefit. For drugs that have a known mechanism of action, biomarkers that relate to this mechanism can be used to measure the effect of the drug on its target. An example of this was done in a phase I study of probenecid and N-acetylcysteine (NAC) in pediatric patients with severe TBI (Hagos et al., 2018). Both drugs are known to increase concentrations of glutathione (an antioxidant) through various mechanisms independently and synergistically. This exploratory trial found that CSF glutathione concentrations were higher in the treatment group than in the placebo group thus providing evidence that these drugs were achieving drug concentrations sufficient to influence glutathione concentrations in the CSF. This confirms the known mechanism of action and encourages further pursuit of this investigational treatment strategy.

If the mechanism of action of a drug is not known, the relationship between a biomarker and a specific pathophysiological pathway or brain component can be used to connect the drug with that pathway. By observing the change in biomarker concentration in response to a drug, one can presume that the drug is impacting that pathway or component. Although not yet explored in clinical trials, preclinical models have suggested this type of relationship. In rats, levetiracetam was found to attenuate the rise in phospho-neurofilament-H (pNF-H) compared to placebo (Yang et al., 2019). The mechanism by which levetiracetam improve outcomes in TBI is not known, but considering pNF-H is specific to axonal damage, it was suggested that levetiracetam diminished axonal injury. Similarly, high-dose valproic acid in swine decreased levels of GFAP and NF-L compared to placebo, which suggests valproic acid might preserve astrocytes and axons after TBI (Korley et al., 2018).

### 2.3 Generalizability of pre-clinical and early clinical outcomes

A significant barrier to TBI drug development is the inconsistent results in preclinical models and human subjects (Kochanek et al., 2020). Large clinical trials could be avoided if data suggested a low likelihood that the drug-disease effect found in preclinical trials would occur in humans. Before expensive clinical phase II/III studies are designed, biomarkers could be used alone or in combination with clinical outcome parameters in smaller and shorter clinical trials to determine the likelihood of a drug achieving the pharmacodynamic effect needed to produce a long-term clinically significant improvement.

The Biomarkers of Injury and Outcome (BIO)-Progesterone for Traumatic Brain Injury, Experimental Clinical Treatment (ProTECT) trial is an example of how biomarkers could have been used to assess the pharmacodynamic effect of a drug prior to a large clinical trial. This trial was performed to investigate the negative results of the ProTECT III trial (Korley et al., 2020; Wright et al., 2014). ProTECT III was a large (N = 882), randomized clinical trial of progesterone versus placebo in patients with moderate to severe TBI. It was designed after preclinical studies suggested neuroprotective effects of progesterone, including decreased cerebral edema and neuronal loss. Although theoretically sound, early administration of progesterone in ProTECT III did not result in improved outcomes at 6 months. As a *post hoc* analysis, BIO-ProTECT used biomarkers [GFAP, UCHL-1, S100β, and spectrin breakdown product (SBDP)150] to demonstrate that progesterone did not decrease brain cell death as desired and suggested that this lack of pharmacodynamic effect in humans may have contributed to the fact that ProTECT III was a negative trial. Other factors including trial design, patient adherence, and patient population certainly contributed to the negative outcome as well, but completion of this large, expensive clinical trial may not have been done if the information from BIO-ProTECT was available prior to beginning ProTECT III.

### 2.4 Early indication of long-term outcomes

TBI is not just an acute injury but often results in long-term impairment that requires chronic follow up. Therefore, clinical trials must demonstrate a prolonged effect beyond the treatment interval on clinical outcomes, which is commonly measured by Glasgow Outcome Scale Extended (GOS-E) at 6 months post-injury. This lengthy follow-up period is susceptible to attrition, thereby requiring more patients to be enrolled to avoid reducing power, which increases cost. Biomarkers measured at shorter intervals (e.g., days to weeks) after injury may decrease the follow-up time needed in clinical drug trials. In the future, if the correlation between biomarker concentrations and long-term outcomes strengthens, clinical trials could strongly suggest long-term benefits of drug treatment by measuring the impact on biomarkers at 24–48 hours rather than conducting lengthy, expensive trials that require 6 months follow-up. Recent studies attempt to demonstrate the connection between blood biomarkers and long-term outcomes, but admit the limitations, including the heterogeneity found among patients with TBI (Yue et al., 2023; Whitehouse et al., 2025; Korley et al., 2022; Crichton et al., 2021; Helmrich et al., 2022; Svingos et al., 2022; Trifilio et al., 2024; Schneider et al., 2023).

### 2.5 Homogenization of study populations and personalization of therapy

The clinical presentation, hospital course and clinical outcomes among patients with TBI are often extremely heterogeneous despite similar presenting GCS scores, imaging, and laboratory results. GCS scores have traditionally been used to classify the severity of a TBI, but this scale is flawed for many reasons including the influence of other factors such as drugs, alcohol, hypotension and hypoxemia. Additionally, GCS does not account for the diverse mechanisms of TBI (e.g., penetrating versus blunt) which more strongly influences clinical intervention and better stratifies patients.

The ability to classify patients into endophenotypes using biomarkers could assist in the development of drugs, particularly monoclonal antibodies, used to target a specific pathophysiology (Wang et al., 2024). Biomarkers can be used to identify which structure of a neuron is damaged or which pathophysiologic mechanism is causing harm. This information may subsequently be used to identify which drugs are most likely to be effective based on its mechanism of action. For example, high concentrations of inflammatory biomarkers may suggest neuroinflammation and the patient may be best treated with immunomodulators. Currently, evidence of targeted and personalized therapy such as this does not exist, which is oftentimes suggested as a reason for the large number of TBI negative trials.

This application of biomarkers would be similar to how monoclonal antibodies (e.g., lecanemab, donanemab) were developed to treat patients with Alzheimer’s disease. For example, donanemab is an amyloid-beta directed monoclonal antibody. Trials of donanemab included only patients with amyloid-beta pathology (Sims et al., 2023). This inclusion criteria increased the likelihood that only patients who would benefit from donanemab would be included. By including only this population, rather than the heterogeneous population of all patients with Alzheimer’s disease, the clinical trial was more likely to be successful. This use is also similar to the identification of genetic mutations in cancer (e.g., HER2, ALK, EGFR) that are targeted by drugs.

Future clinical trials may use biomarkers to select a homogenous patient population likely to benefit from the drug intervention; so far, biomarkers have been used to justify drug studies based on mechanism of action and type of injury. For example, one group chose to study glyburide, a sulfonylurea receptor 1 (SUR1) antagonist, because CSF concentrations of SUR1 predict swelling and outcome in patients with TBI (Eisenberg et al., 2020). To increase the likelihood that patients enrolled in a future trial of glyburide would benefit from SUR1 antagonism, use of SUR1 concentrations as inclusion criteria could be considered. However, the practical implications of obtaining hyperacute CSF sample analysis and results prior to enrollment would first need to be addressed. Similarly, a study in rats suggested that levetiracetam diminished axonal injury because concentrations of pNF-H were lower in the drug treatment group than in the placebo group (Yang et al., 2019). This evidence could be used to justify a study of levetiracetam in patients with evidence of diffuse axonal injury after TBI with the hope that the endophenotype of TBI most likely to benefit was chosen.

## 3 Conclusion

As a growing component of TBI research, innovative methods will be employed to incorporate biomarkers into preclinical and clinical trial design. Key areas for research include using population biomarker kinetics to determine when it is best to start drug therapy in a clinical trial, initiating or discontinuing drug treatment as a reaction to a rise or fall in individual biomarker concentrations, determining a biomarker threshold as an inclusion or exclusion criterion, and identifying when a secondary injury is occurring using biomarker concentrations and subsequently intervening at that point.

The use of biomarkers to influence TBI drug development and treatment trials has the potential to lead to more innovative research and personalized patient care. In the future, strong correlations between biomarkers and clinical outcomes could permit shorter, less expensive, and more successful clinical trials.
